# Incidence and predictors of first-year unplanned discontinuation of Implanon at Ayder comprehensive specialized hospital, northern Ethiopia: A retrospective follow-up study

**DOI:** 10.1371/journal.pone.0259234

**Published:** 2022-01-26

**Authors:** Hiluf Ebuy Abraha, Kebede Embaye Gezae, Alemayehu Bayray Kahsay, Mengistu Hagazi Tequare

**Affiliations:** 1 College of Health Science, Ayder Comprehensive Specialized Hospital, Clinical Governance and Quality Improvement unit, Mekelle University, Tigray, Ethiopia; 2 College of Health Sciences, School of Public Health, Mekelle University, Tigray, Ethiopia; Jhpiego, UNITED STATES

## Abstract

**Background:**

Discontinuing contraception without switching to a different type of family planning (FP) method contributes to unwanted pregnancy and unsafe abortion. Unplanned discontinuation of Implanon (which is discontinuation of Implanon without switching, but not for reasons of wanting to get pregnant) during the first year and its possible determinants have not been well investigated in Ethiopia. Therefore, this study aimed to determine the incidence and predictors of unplanned discontinuation of Implanon during the first year.

**Methods:**

A retrospective follow-up study was conducted among 413 consecutive series of eligible women at Ayder Comprehensive Specialized Hospital in Northern Ethiopia. Data were drawn from both FP initiation and removal registration books and from contacting users by phone over a one-year period (April 2016 and March 2017). The inclusion of the categorical predictor in the final Cox model was considered if the test had a P-value of <0.25 in the log-rank test. We identified predictors of time to unplanned discontinuation using a multivariable Cox regression analysis. Adjusted hazard ratios with 95% confidence intervals (CI) were used to assess the association of covariates with the risk of discontinuation. There were no statistically significant interaction terms and proportionality assumption was fulfilled.

**Results:**

The unplanned discontinuation rate of Implanon during the first year was 18.2%, with an incidence density of 16.3 discontinuations/1000 women-months. Compared with those under 20 years of age, women aged 20 to 24 years (AHR = 0.42; 95% CI: 0.19–0.91) had a protective effect against discontinuation. On the other hand, clients whose partner’s educational level was lower than secondary (AHR = 2.20; 95% CI: 1.08–4.49) and who had never used any modern contraception method before (AHR = 3.26; 95% CI: 1.61–6.61) had a higher risk of discontinuation.

**Conclusions:**

Our findings have significant implications for understanding why Implanon is discontinued in an unplanned manner, and will help policy makers plan the interventions needed to improve Implanon continuity by overcoming identified barriers. Providers in similar settings should pay more attention to clients whose partners have lower educational status and who are new acceptors.

## Background

Although not all discontinuations are necessarily problematic, as family planning (FP) programs expand and access increases, unnecessary and undesirable discontinuation without switching to a different type of contraceptive is a significant challenge. The odds of discontinuation of modern contraceptives vary widely by method, particularly long-acting versus short-acting [1,2]. Implanon, despite its effectiveness and improvement in use, is discontinued by a proportion of users before its expiration date (3 years). Reports have shown that access to FP methods differ significantly by region [3]. However, regarding the discontinuation of the first year of Implanon, a similar magnitude was observed between developed and developing countries, ranging from 13.5% to 28% [4–7].

The 2019 Ethiopia Mini Demographic and Health Survey (EMDHS) revealed that modern contraceptive use has tripled in the past 14 years. Implants accounted for only 9.0% of modern contraceptives, although they are the most commonly used methods among long-acting reversible contraceptives and their use has increased dramatically from just 0.3% in 2005 to 8.0% in 2016. Among implants, Implanon is the most commonly used method in Ethiopia [8–10]. However, a significant number of women are discontinuing their use of Implanon within one year of use. Studies from Ethiopia showed that the discontinuation rate of Implanon during the first year ranged from 16% to 23.9% [11–13].

Different contributing factors can affect the discontinuation of the first year of Implanon. Age, marital status, prior contraceptive use, partner educational level, counselling on side effects, and parity are among the predictors reported in developed and developing countries [4,7,11,14,15]. A report showed that contraceptives are estimated to prevent nearly half of maternal deaths [16], and if stopped early without switching or for reasons other than wanting to get pregnant, it contributes to unwanted pregnancies, miscarriages, and can lead to pregnancies that may be terminated through unsafe abortion [2,17].

Service delivery capacity for implant removals moves at a slower pace compared to insertion [18]. In Ethiopia, until 2009, Health Extension Workers (HEW) could only provide injectables. However, in 2009, they were trained on the insertion of Implanon as a means of the Implanon expansion program in the country. Because HEWs cannot remove Implanon, the program provided implant removal training to other healthcare providers [8].

In Ethiopia, different researchers [11–13,19] identified the rates and factors associated with discontinuation of Implanon. However, almost all [12,13,19] of these studies focused on looking at possible factors affecting discontinuation of Implanon at its near expiration date. Furthermore, in all the previous studies [11–13,19] planned discontinuations, such as removals due the desire to become pregnant and to have children, were considered as an event of interest. To date, there is insufficient evidence regarding the magnitude of the unplanned discontinuation of Implanon in its first year of use and its possible contributing factors have not been well examined. Therefore, this study was designed to partly fill the aforementioned gap and contribute its part by determining the incidence and predictors of unplanned discontinuation of Implanon during the first year at Ayder comprehensive specialized hospital (ACSH), Mekelle, Tigray, Ethiopia.

## Materials and methods

### Study area and period

This study was conducted at ACSH, which is located in Mekelle, Tigray, Ethiopia. The College of Health Sciences, ACSH is a public hospital with a capacity of 650 beds [20]. Implanon is provided at three sites of the hospital. The first site is in the labour ward; which is given to immediate postpartum mothers and the second is given in the gynecology ward for women in their immediate post-abortion period. Finally, for interval insertions, Implanon is given at an on-campus clinic, called Michu clinic. However, removal of Implanon for all primers is done at that clinic. The study was conducted from December 2019 to July 2020, using data between April 2016 and March 2017.

### Study design and population

We employed a hospital-based retrospective follow-up study design. The study population was all women of reproductive age who initiated their Implanon at ACSH between April 2016 and March 2017 and who had removal data in the registries.

### Eligibility criteria

Women who had an Implanon inserted between April 2016 and March 2017 at the ACSH were included in our study. Users whose timing of insertion or discontinuation of Implanon was not documented were excluded.

### Sample size determination and sampling method

For the incidence of discontinuation of Implanon during the first year, the minimum sample size was calculated from an earlier study conducted in Ofla, Tigray, Ethiopia [13] using the formula for a single population proportion. The study proportion of the discontinuation rate of Implanon during the first year was 16%. Where, Z_α/2_ = 1.96, margin of error = 0.04, p = proportion of the rate of discontinuation of Implanon in the first year of the previous study, and q = 1-p.


$$\text{Sample}\;\text{size}(\text{n})=\frac{\left(Z\alpha /2\;\right){\;}^{2}\;*p*q}{\left(d\right){\;}^{2}}=323$$

For predictors, the minimum sample size was estimated from the previous study that was focused on predictors of Implanon discontinuation in the first year conducted in Egypt [7]. The calculation of the sample size for the survival analysis [21] was considered based on the following underlying assumptions: two population proportions (q0 = proportion of the unexposed group and q1 = proportion of the exposed group), statistical power of 90%, 95% CI, and one-year discontinuation probability and hazard ratio from the previous study. Considering the previous use of Implanon as an exposure variable (previously used as an exposed and not used as unexposed). Where, q0 = proportion of unexposed Implanon users = 66.5%, q1 = proportion of exposed Implanon users = 33.5%, Hazard ratio (HR) = 0.36, Z_α/2_ = 1.96, Z_β_ = 1.282, Probability of one-year discontinuation of Implanon P(E) = 13.5%.


$$\text{Number}\;\text{of}\;\text{events}\;\text{needed}\;\left(\text{E}\right)=\frac{\left(Z\alpha /2+\;Z\beta \right){\;}^{2}}{\left(Log\;HR\right){\;}^{2}\;*qo*q1}=45$$

$$\text{Sample}\;\text{size}\;\left(\text{n}\right)\frac{E}{P\left(E\right)}=335$$

To maintain the optimal statistical power of 90%, the minimum sample size (335) would have been used. However, 413 user records that started Implanon from April 2016 to March 2017 were reviewed and included consecutively. The time period, April 2016 to March 2017, was set for users to complete the full three years Implanon use period.

### Sampling procedure

**Fig 1:**
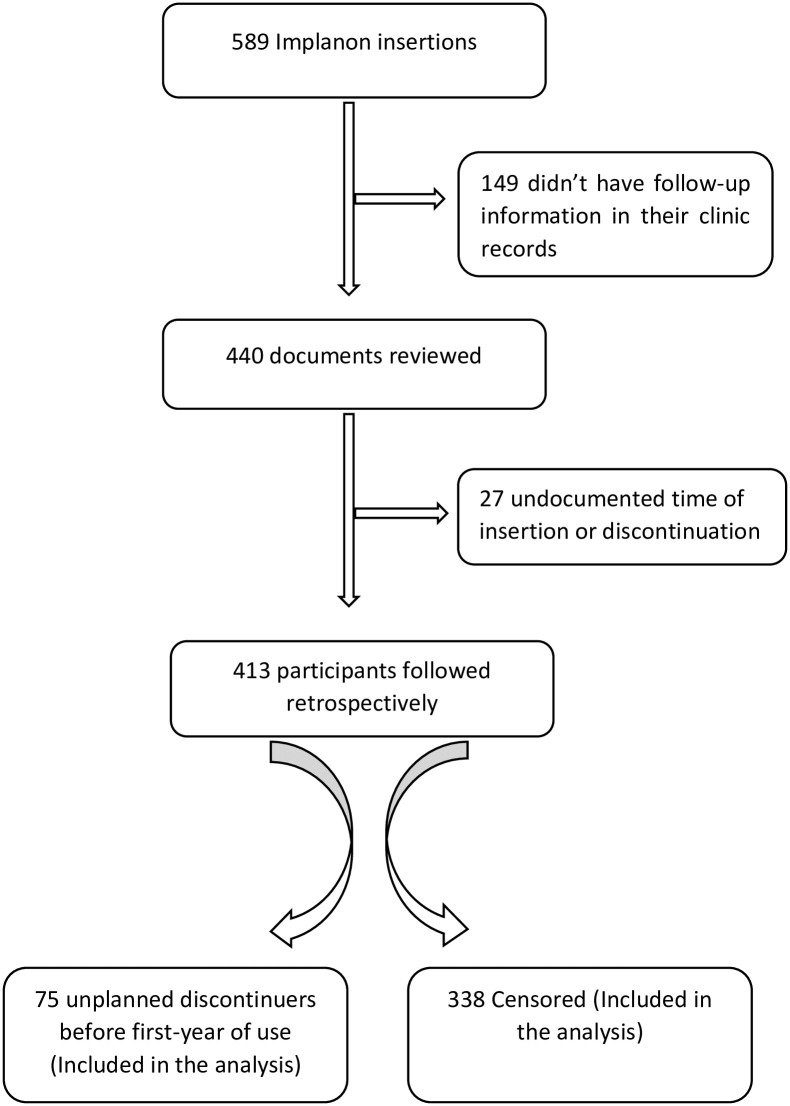
Flow of the study population illustrating the eligibility for inclusion in the analysis of unplanned discontinuation of Implanon at Ayder comprehensive specialized hospital, Mekelle, Northern Ethiopia, 2016–2017 (n = 413).

There were a total of 589 initiators between April 2016 and March 2017. Although 149 (25.3%) of them were excluded because they had no follow-up information in their clinical records, we excluded 27 (4.6%) of them as time of insertion or discontinuation was not documented (Fig 1). Those who did not have follow-up information in their records did not differ from those who were included in the analysis by age, residence and prior contraceptives exposure (data not shown).

### Outcome definition and measurements

#### Unplanned discontinuation of Implanon

Evidence suggests that discontinuation is not always negative and is predictable. Discontinuation is a problem when women do not immediately switch to another method while not planning to become pregnant, which can put them at risk for an unintended pregnancy [2]. Therefore, unplanned discontinuation of Implanon is the discontinuation of Implanon without switching; it could be due to health concerns, misconception (myths and misunderstandings about contraceptives use), partner influence, or contraceptive failure, but not due to reasons of planned removals such as wanting to get pregnant.

#### Time to unplanned discontinuation

Length of follow-up time, i.e., from the insertion of Implanon to its discontinuation within the first year of initiation. Time to discontinuation, which was measured in months, was treated as a continuous variable, with a maximum time allowed of 12 months.

### Data collection tools and procedure

We used a questionnaire and a checklist to collect the data (S1 and S2 Tables). Initially, using a checklist, we extracted and collected the data by reviewing the FP initiation and removal registration books retrospectively. Study participants were selected from FP initiators registration book. We reviewed the registration books up to three years after device placement to confirm a one-year continuation status. This was done because not all users had a clinic visit one year after device placement. For example, if a client did not show up at the first-year of use but had a visit within two-years of insertion and the registration book indicate that the device was discontinued, she would be classified as a continuer in the first year. Among the discontinuers, the reasons for removal were recorded as side effect, misconceptions, and others; which included partner influence, partner death and contraceptive failure.

### Phone survey

After extracting the necessary secondary data, a separate questionnaire was used for the phone interview. We telephoned all (413) users only for possible sociodemographic predictors (user’s educational level, marital status, user’s occupation, partner’s educational level and partner’s occupation), which were not recorded in the secondary data sources. All users had phone information. Three attempts, which were made on different days during the data collection period, were made to reach users whose phone was not working. We interviewed them for an average of three minutes. The overall response rate to the telephone call was 364 (88.1%). All those who did not respond were women who had a telephone but could not be reached. The discontinuers and continuers had a fairly similar response rate for the phone call, with 86.7% and 88.5% respectively. However, the 11.9% of those who did not respond were included in the analysis for a couple of reasons; first, in order to reduce a bias that can systematically exclude those without phones. Second, all users had a complete record of the outcome variable.

Non-response bias is a threat when the response rate falls below 30% and its possible occurrence can be assessed by comparing the characteristics of those who respond and those who do not respond [22]. Accordingly, a chi-square test for association was checked and showed that those who were and were not contacted did not differ in terms of basic characteristics such as, age, residence, side effect, duration of Implanon use, and discontinuation status (data not shown). The time of insertion was between April 2016 and March 2017 but the phone interview was in 2020, at least three years after insertion. So, when we were in contact with the women, we asked them about their sociodemographic status during the insertion period.

#### Study variables

Our outcome variable was the time to unplanned discontinuation of Implanon during the first year. Consequently, the event was labeled as 1 (discontinuer) and 0 censored (removal after one year of use, switching after discontinuation or removal due to desire to get pregnant).

Sociodemographic factors (age, marital status, residence, user’s occupation, partner’s occupation, user’s educational level, partner’s educational level), reproductive health characteristics (parity, HIV status), factors related to contraceptives (previous exposure to contraceptives, side effects, contraceptive failure), and factors related to the initiation period (immediate postpartum period, post-abortion, interval) were the independent variables.

### Data quality assurance and control

The data extraction checklist was prepared in English based on the variables obtained from different literatures [5–7,9,12]. The questionnaire containing the questions to be asked via cell phone was translated into Tigrigna, which is the local language of the study area. Then, it was translated back into English for consistency. A preliminary test was performed at 5% of the sample size at a nearby health center named Mekelle Hospital.

Training was provided on the checklist, data collection technique, purpose of the study, and maintaining confidentiality for data collectors. The data collectors were subsequently monitored for any concerns regarding the data.

### Data management and analysis

We encoded and entered the data into EpiData version 4.6; it was then exported to Stata version 15 statistical software for analysis. Data were checked for consistency and completeness, and cleaned and edited prior to analysis.

#### Descriptive statistics and categorical data analysis

Discontinuation rates at different times and reasons for interruptions were analyzed using frequency and percentage. The incidence rate was reported as the number new discontinued cases per 1000 women-months of observation. We used a log-rank test to select potential candidate categorical predictors. Kaplan-Meier curve estimates of the probability of discontinuation were plotted to distinguish the median time to unplanned discontinuation of Implanon and to test the significance of observed differences between strata.

#### Model building

Predictors of time to unplanned discontinuation were identified using a multivariable Cox proportional hazards model. Adjusted hazard ratios (AHR), 95% CI and P-values were calculated to assess the association of various determinants with the risk of discontinuation. A final Cox proportional hazard model was fitted by including all potential predictors with a significance level <0.25 in the log-rank test. A P-value less than 0.05 was considered statistically significant. We checked all the possible interactions and found no statistically significant interaction terms.

#### Proportional hazard assumption

Proportionality was checked by including time-dependent covariates in the model, and the interaction between covariates and time was not statistically significant. This implies that the effect of a risk factor is constant over time and therefore, proportionality assumption of the Cox hazards model was fulfilled [23].

The ratio of the hazard function (h_(t)_) to the baseline hazard function (ho_(t)_) at any time is expected to be constant. The Cox proportional hazards model assumes that the predictors have a multiplicative effect on the hazard and that this effect is constant over time. In the survival function, the Kaplan Meier (KM) curves will approximately parallel to each other.

Baseline model when all predictors are zero;

$$\text{H}\left(\text{t}\right)={\text{h}}_{\text{o}}\left(\text{t}\right)$$

Main effect model with coefficients;

$$\text{ln}\;\text{h}\left(\text{t}\right)=\text{ln}\;{\text{h}}_{\text{o}}\left(\text{t}\right)+\beta_{1}{\text{X}}_{1}+\beta_{2}{\text{X}}_{2}\ldots \ldots +\beta_{\text{p}}{\text{X}}_{\text{p}}\;\text{OR}\;{\text{ln}}^{\;}\;\frac{h\left(t\right)}{ho\left(t\right)}=\beta_{1}{\text{X}}_{\text{1}}+\beta_{2}{\text{X}}_{\text{2}}\ldots \ldots +\beta_{\text{p}}{\text{X}}_{\text{p}}$$

[24]

#### Model diagnostics and fitness

The goodness of fit of the final Cox model was determined using martingale residuals with “stcoxgof” command. The model gave a P-value of 0.21, which shows our final Cox model is a good model.

### Ethical considerations

Ethical clearance was obtained from the Institutional review board of the college of health sciences of the university of Mekelle (review reference ERC1520/2020). After informing the purpose and procedure of the study, verbal consent was taken if participants were willing to give their sociodemographic characteristic through a phone call. Their right not to participate, not to answer any questions and to withdraw from the interview at any time they wanted was respected.

## Results

### Sociodemographic characteristics

A total of 413 women were included in the study. The age of the participants ranged from 15 to 48 years with a median of 25 years and Interquartile Range (IQR) of 9 years. One hundred twenty-three (29.8%) of them were between 20 and 24 years old. The majority (85.2%) of the participants were urban dwellers. Among the women contacted, more than half (56.9%) of them had a secondary or higher level of education. Three-quarters (74.5%) of the telephoned users were married, of whom 167 (61.6%) the educational status of their partners was secondary or higher. Regarding occupation, while 164 (45.0%) of the study participants were housewives, 126 (46.8%) of the partners were government employees (Table 1).

**Table 1:** Baseline sociodemographic characteristics of the study participants at Ayder comprehensive specialized hospital, Mekelle, Northern Ethiopia, 2016–2017 (n = 413).

Variable	Category	Frequency (%)	Number of women-months observation
**Age category (Years)**	<20	63 (15.2)	675
	20–24	123 (29.8)	1387
	25–29	113 (27.4)	1301
	30–34	61 (14.8)	684
	>34	53 (12.8)	551
**Residence**	Urban	352 (85.2)	3963
	Rural	61 (14.8)	635
**User educational status (n = 364)**	≥ Secondary education	207 (56.9)	2339
	< Secondary education	157 (43.1)	1713
**Marital status (n = 364)**	Single	53 (14.4)	591
	Married	271 (74.5)	3008
	Divorced	36 (10.0)	411
	Widowed	4 (1.1)	42
**If married, partner educational status (n = 271)**	≥ Secondary education	167 (61.6)	1898
	< Secondary education	104 (38.4)	1110
**User occupation (n = 364)**	House wife	164 (45.0)	1814
	Government employee	63 (17.3)	700
	Private/NGO	74 (20.3)	841
	Student	46 (12.7)	524
	Farmer	9 (2.5)	90
	Jobless	8 (2.2)	83
**If married, partner occupation (n = 271)**	Government employee	126 (46.8)	1444
	Private/NGO	97 (36.0)	1061
	Farmer	30 (11.2)	325
	Guard	8 (3.0)	84
	Daily laborer	8 (3.0)	94

### Reproductive and contraception related characteristics

Users started Implanon at different periods of insertion; namely, interval, postabortion, and immediate postpartum. Consequently, nearly half (46.2%) of them were in the interval period and 140 (33.9%) were in their immediate postpartum period. One hundred fifty-six (39.8%) of them had previously used modern contraceptives at least once. Furthermore, 306 (97.1%) of the participants were HIV negative. Of the individuals, 152 (40.3%) of them have had more than one child (Table 2).

**Table 2:** Reproductive and contraception related characteristics of the study participants at Ayder comprehensive specialized hospital, Mekelle, Northern Ethiopia, 2016–2017 (n = 413).

Variable	Category	Frequency (%)	Number of women-months observation
**Period of insertion**	Interval	191 (46.2)	2114
	Immediate postpartum	140 (33.9)	1573
	Post abortion	82 (19.9)	911
**Previous exposure to modern contraceptive method (n = 392)**	Yes	156 (39.8)	1806
	No	236 (60.2)	2557
**HIV status (n = 315)**	Positive	9 (2.9)	103
	Negative	306 (97.1)	3406
**Parity (n = 377) (range, 0–9)**	Nulliparous	132 (35.0)	1468
	Para one	93 (24.7)	1049
	Multiparous	152 (40.3)	1680

#### Reasons for unplanned discontinuation

**Fig 2:**
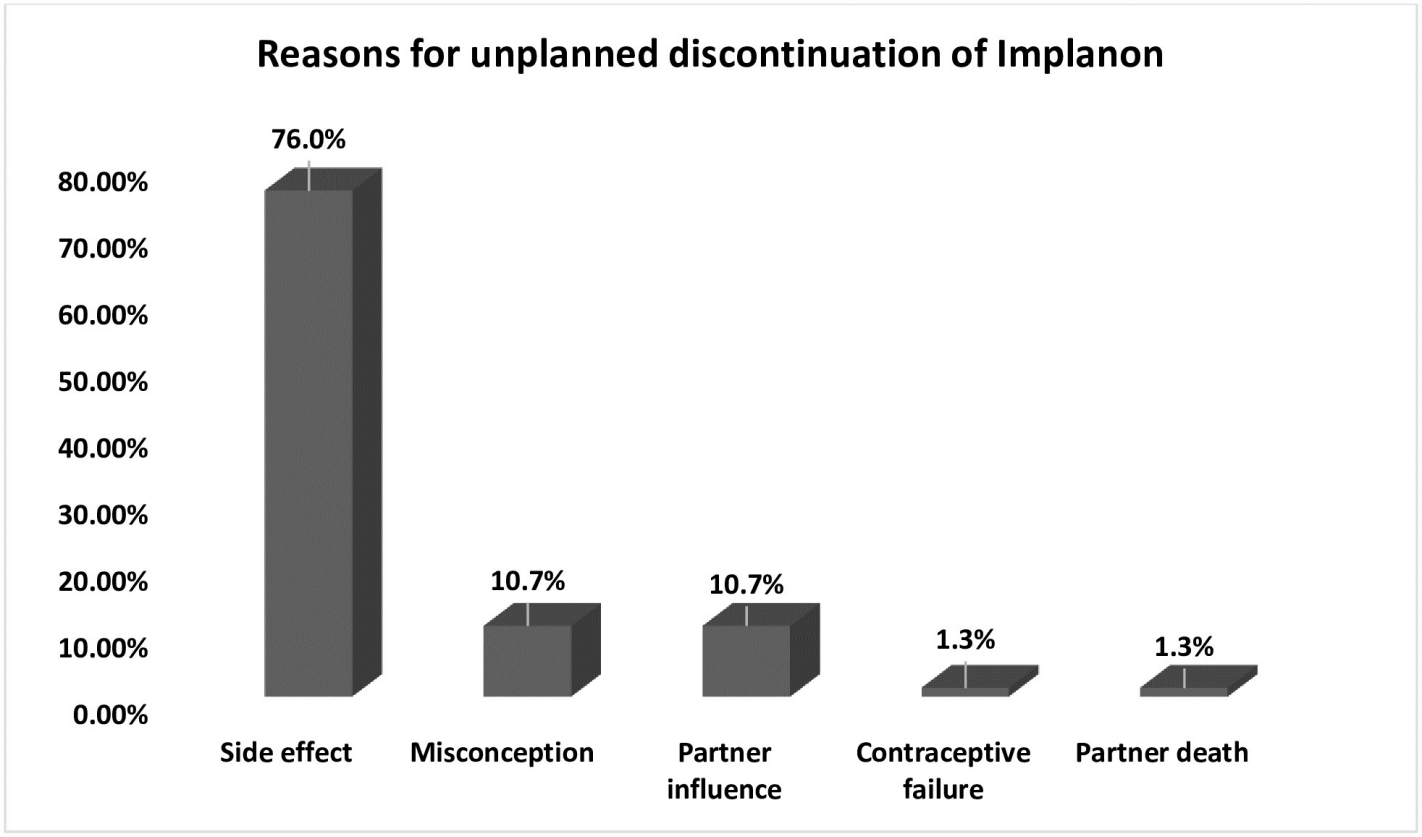
Reasons for the unplanned discontinuation of Implanon at Ayder comprehensive specialized hospital, Mekelle, Northern Ethiopia, 2016–2017 (n = 413).

The main reasons cited for the unplanned discontinuation of Implanon during the first year were: side effect 57 (76.0%), misconception 8 (10.7%), partner influence 8 (10.7%), and others (contraceptive failure and death of the partner), which accounted for 2 (2.6%) (Fig 2).

### Incidence of first-year unplanned discontinuation of Implanon

The minimum duration of use was three months. The unplanned discontinuation rate of Implanon was 4.8% (95% CI: 3.0–7.4) at six months and 18.2% (95% CI: 14.6–22.2) at twelve months. The total study participants were followed and observed for 4598 women-months. At the end of the 12^th^ month, 75 (18.2%) of the 413 individuals discontinued, providing an overall incidence rate of 16.3 (95% CI: 13.0–20.4) discontinuations per 1000 women-months. Due to the low number of unplanned discontinuers, the median time to unplanned discontinuation could not be determined. But, among the discontinuers the median time to device removal was 9 months with IQR of 6–10.

**Fig 3:**
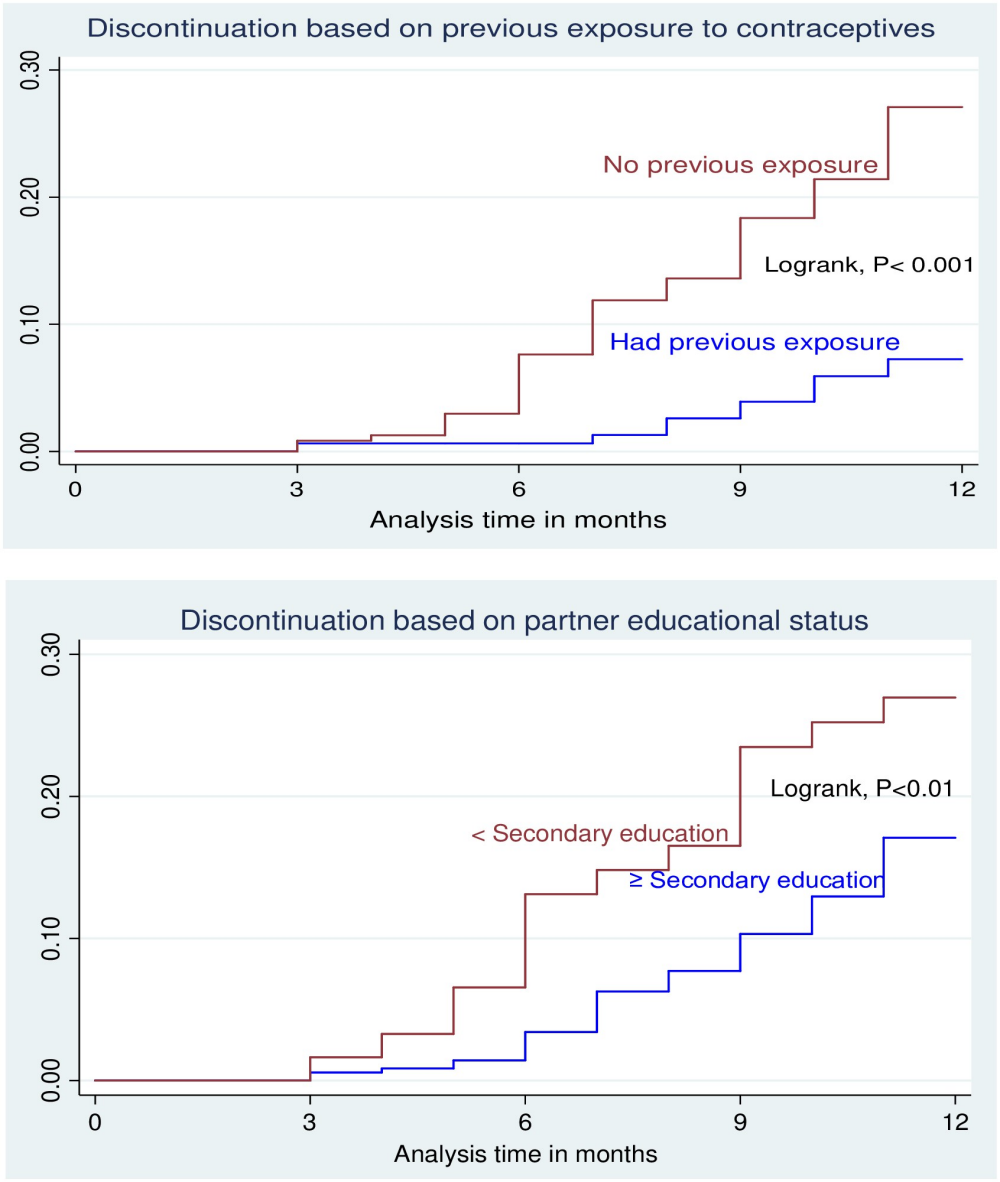
Incidence of unplanned discontinuation of Implanon based on previous exposure to contraceptives and partner educational status at Ayder comprehensive specialized hospital, Mekelle, Northern Ethiopia, 2016–2017 (n = 413).

The cumulative incidences (risks) among users whose partner had secondary or a higher level of education was 13.8%, while among those whose partner had an educational level below secondary school the risk was 28.8%. Furthermore, users who had an experience in the use of modern contraceptive had a cumulative incidence of 7.0% while those who had no previous experience had a cumulative incidence of 26.7% (Fig 3).

### Predictors of first-year unplanned discontinuation in the final Cox model

Eight covariates were included in the final Cox regression model, and the variables age of the participants, educational level of the partner, and previous exposure to contraceptives were predictors significantly linked to the hazard of unplanned discontinuation of Implanon. Thus, the risk of an unplanned discontinuation rate of Implanon was reduced by 58% (AHR = 0.42; 95% CI: 0.19–0.91) among those who were in the age category of 20 to 24 years compared to those younger than 20 years. Clients whose partner’s educational level was lower than secondary were more likely to discontinue Implanon during the first year of use compared to those whose partners had secondary or a higher level of education (AHR = 2.20; 95% CI: 1.08–4.49). Furthermore, women who never used any modern contraceptive method in the past had a 3 times higher risk of discontinuation of Implanon in the first year compared to their counterparts (AHR = 3.26; 95% CI: 1.61–6.61) (Table 3).

**Table 3:** Cox regression analysis for predictors of unplanned discontinuation of Implanon at Ayder comprehensive specialized hospital, Mekelle, Northern Ethiopia, 2016–2017 (n = 413).

Variables	Category	CHR (95% CI)	AHR (95% CI)	P-Value
**Age category**	<20	1.00	1.00	
	20–24	0.47(0.24–0.91)	0.42(0.19–0.91)	**0.029**
	25–29	0.36(0.18–0.74)	0.47(0.19–1.17)	0.110
	30–34	0.68(0.34–1.40)	0.83(0.36–1.93)	0.670
	≥35	0.90(0.44–1.84)	0.36(0.10–1.31)	0.120
**Residence**	Urban	1.00	1.00	
	Rural	1.92(1.12–3.29)	1.30(0.37–4.62)	0.680
**User educational status**	≥ Secondary education	1.00	1.00	
	< Secondary education	1.95(1.19–3.19)	1.49(0.76–2.95)	0.250
**Partner educational status**	≥ Secondary education	1.00	1.00	
	< Secondary education	2.29(1.33–3.95)	2.20(1.08–4.49)	**0.029**
**User occupation**	Government employee	1.00	1.00	
	Jobless	2.19(0.46–10.33)	0.56(0.06–5.56)	0.620
	Private/NGO	1.11(0.44–2.76)	1.77(0.51–6.07)	0.360
	Student	0.81(0.27–2.49)	0.69(0.12–4.07)	0.690
	Farmer	3.14(0.83–11.83)	0.98(0.18–5.26)	0.980
	House wife	1.72(0.80–3.70)	1.44(0.51–4.09)	0.490
**Partner occupation**	Government employee	1.00	1.00	
	Daily laborer	1.39(0.32–5.92)	0.57(0.11–2.91)	0.500
	Private/NGO	1.10(0.58–2.09)	0.69(0.33–1.47)	0.340
	Farmer	2.71(1.33–5.51)	0.95(0.23–3.89)	0.950
	Guard	0.86(0.12–6.42)	0.57(0.06–5.19)	0.620
**Period of insertion**	Post abortal	1.00	1.00	
	Immediate postpartum	0.45(0.22–0.91)	0.66(0.27–1.62)	0.360
	Interval	1.04(0.60–1.80)	1.54(0.74–3.20)	0.250
**Previous exposure to contraceptives**	Yes	1.00	1.00	
	No	4.17(2.19–7.91)	3.26(1.61–6.61)	**0.001**

1.00: Reference Category, CHR: Crude Hazard Ratio, AHR: Adjusted Hazard Ratio, CI: Confidence Interval.

Due to the incompleteness of data for some variables (user and partner educational status and user and partner occupation) imputation was performed before the analysis. However, no difference in the significance of the predictors was observed even when the Cox regression analysis was run while the variables are missing. The final Cox model was, therefore, performed without any imputation, so the results should be interpreted with caution.

## Discussion

This study was conducted to determine the incidence and predictors of unplanned discontinuation of Implanon during the first year. Consequently, among those who had their implanon inserted, the overall incidence rate was 16.3 (95% CI: 13.0–20.4) discontinuations per 1000 women-months with 18.2% (95% CI: 14.6–22.2) unplanned discontinuation rate. The age of the participants, the educational level of the partner, and previous exposure to contraceptives were statistically significant predictors of unplanned discontinuation of Implanon during the first year.

We found that 18.2% (95% CI: 14.6–22.2) of the users discontinued their Implanon during the first-year of use, with an overall incidence density of 16.3 (95% CI: 13.0–20.4) discontinuations per 1000 women-months. The unplanned discontinuation rate of Implanon during the first year was comparatively lower than studies conducted in Australia, Netherlands, and a couple of reports from Ethiopia [5,6,11,12]. In contrast, the rate in our study was slightly higher compared to a study conducted in Egypt [7]. However, the figure was similar with reports from the USA, Nigeria, Kenya, and Ethiopia [4,13,25–27]. The low rate of discontinuations in our study could be related to the fact that in comparative studies, discontinuations such as removal due to wanting to get pregnant were considered as an event of interest. However, in our case, 2.2% of the total users or 9.6% of the first-year discontinuers who removed their Implanon for reasons that they wanted to become pregnant were excluded from the numerator, which might have underestimated the discontinuation rate in the current study. Possible variation in service delivery settings could also be the reason for the difference in discontinuation rates between the various studies, given that our study was conducted in a large but single hospital, while the others were either multicentered, community or small pocket reports.

It can be seen from the data in Table 3 that the risk of unplanned discontinuation of Implanon among users in the age categories of 20 to 24 years was lower than that of those under 20 years of age. This finding supports evidence from previous cross-sectional study conducted in southern Ethiopia, which showed women aged 20 to 24 years, 25 to 29 years, and older than 35 years had a protective effect against discontinuation compared to those under 20 years of age [11]. However, in reports from Australia and Egypt, age was not a statistically significant predictor of the first-year Implanon discontinuation, in the final Cox regression model [6,7]. There are a number of plausible explanations for the discrepancy: first, it could be due to the fact that the majority (72.39%) of the participants were young, under 30 years of age. Second, our study did not include covariate counselling prior to insertion as a predictor. Lastly, the difference in the time period in which the study was conducted could also be another reason for the discrepancy. In contrast, in comparative studies either the majority of their participants were over 30 years of age or pre-insertion counselling was an integral part of the study. A greater understanding of the Implanon discontinuation dynamics among women of different age groups could be gained through a more comprehensive look at the reasons for discontinuation and at the relationship between Implanon use and marital status.

Another important finding was, that clients whose partner educational level lower than secondary were more likely to discontinue Implanon during the first year of use compared to users whose partners had secondary or a higher level of education. This finding was in agreement with a similar study conducted in Egypt [7]. Which can be attributed to the support of educated husbands for the idea of having a small family size and, therefore, encouraging the use of FP [28]. In addition, educated partners may have a better knowledge and awareness of the benefits of contraceptives and understand when side effects occur. To further explain, our finding showed that among the reasons cited for unplanned discontinuation of Implanon during the first year, partner influence accounted for 8 (10.7%) of all reasons, of whom 6 (75.0%) of the partners educational level was lower than secondary. This, presumably, indicates that less educated partners influence their spouses to stop using their device early. In general, partner’s perspectives and opinions play an important role in contraceptives discontinuation decisions [2]. However, interventions have indicated that if we made men aware of the economic advantages of FP, they could be more supportive continued use by their wives [29,30]. Furthermore, a report in Bangladesh showed that counselling husbands regarding contraception was associated with higher rates of implants continuation [31]. Ultimately, this can lead to narrowing the knowledge gap and contraceptive myths, especially among less-educated partners, thereby increasing continuation rates.

Discontinuation of Implanon during the first year was higher among users who never used any modern contraception method in the past. This finding broadly supports the work of a study from Egypt which revealed that clients who had previously used Implanon were less likely to discontinue their current Implanon during the first-year of use [7]. One explanation for this result may be that women who accepted the contraception side effects during previous use tend to continue using their current device. In contrast, a study from the USA found that women who had previously used oral contraceptives, intrauterine device (IUD), combined hormonal contraceptives, and other methods were more likely to discontinue their etonogestrel implants at first-year of use [14]. This contradictory finding could be due to the difference in the types of contraceptive methods used during the baseline period of the two studies; in the US report, previous use of contraceptives included methods that were not available in our setting, such as the patch and the vaginal ring. However, a broad focus on the specific type of previous method uses and its effect on the continuation status can help us verify the clear impact of the methods.

Our study focused on unplanned discontinuers of Implanon, which doesn’t include neither switchers nor discontinuers for reasons of wanting to get pregnant. Because, in developing countries half of all unplanned pregnancies are terminated, most of which are take place in unsafe settings [17]. Our findings have significant implications for the understanding why women discontinue their Implanon in unplanned way. And, it will help programmers, FP leaders, and policy makers to plan the interventions needed to improve the continuity of Implanon use by overcoming identified barriers to unplanned discontinuations.

### Strength of the study

Notwithstanding the retrospective study, this work offers valuable insights on the incidence density of unplanned discontinuation. The study certainly adds to our understanding of the possible predictors of unplanned discontinuation of Implanon during the first year. Furthermore, unlike most of the previous studies, which focused on interval, post abortion or immediate postpartum periods, our study took into account all three insertion periods.

### Limitations of the study

This study has several limitations. First, 29.88% of users were excluded from the study, which is a potential source of bias. The retrospective nature of our study made it impossible to investigate some factors related to provider (skill of the service provider and pre-insertion counselling status) and the health system (health facility setup). Furthermore, most of the participants reasoned that side effects were the main cause of the unplanned discontinuation, but the specific type of side effect was not recorded. Incomplete data due to the retrospective nature of our study design might also have affected our work to some degree. Also, when users were phoned, they might have encountered a potential recall bias.

## Conclusions

Almost one fifth of users, which is lower than most previous studies, discontinued using their Implanon in unplanned way during the first-year. The more significant finding emerged from this study was that age of the participants, the educational level of the partner, and previous exposure to contraceptives were statistically significant predictors of unplanned discontinuation of Implanon during the first year. Providers in a health facility setting should pay more attention to women whose partners have lower educational level, and who are new acceptors. Also, more male involvement should be practiced during method insertion. A further study could assess the differences between discontinuation of Implanon and discontinuation of other FP methods or discontinuation in the first-year and discontinuation in other years.

## Supporting information

S1 Table English version questionnaire and checklist. (DOCX)

S2 Table Tigrigna (local language) version questionnaire used for the phone survey. (DOCX)

S1 Dataset (DTA)
