# Supplementary material for: Incidence and predictors of first-year unplanned discontinuation of Implanon at Ayder comprehensive specialized hospital, northern Ethiopia: A retrospective follow-up study
Source: PLoS One. 2022 Jan 26;17(1):e0259234. doi: 10.1371/journal.pone.0259234 (PMC8791466; doi:10.1371/journal.pone.0259234)
Supplement: S1 Table — (DOCX) [file pone.0259234.s001.docx]

**Data collection sheet (English Version)**

**Part I. Woman’s sociodemographic characteristics (phone survey)**

You were one of the users who Implanon was inserted between April 2016 and March 2017. The following sociodemographic related questions should, therefore, show your characteristics when the device was inserted.

| Serial no. | Question | Response | Skip rule |
| --- | --- | --- | --- |
| 101 | What was your educational status? | 1. No formal education 2. Primary school 3. Secondary school 4. College/university |  |
| 102 | What was your marital status? | 1. Single 2. Married 3. Divorced 4. Widowed | If not married, skip to 104 |
| 103 | If you’re married, what was your husband’s educational status? | 1. No formal education 2. Primary school 3. Secondary school 4. College/university |  |
| 104 | What was your occupation? | 1. Daily laborer 2. Government employee 3. Private/NGO 4. Student 5. Farmer 6. Housewife 7. Other (Specify) |  |
| 105 | If you’re married, what was your husband’s occupation? | 1. Daily laborer 2. Government employee 3. Private/NGO 4. Farmer 5. Other (Specify) | Skip if not married |

NGO: Non-Governmental Organization

| Serial number | MRN (Medical Record Number) | Age in years | Residence (1=urban, 2=rural) | HIV status (1=+ve, 2= -ve) | Parity | Previous exposure to contraceptives (1=yes, 2=no) | | Period of insertion | Date of Implanon insertion | Date of Implanon removal | Duration of use in months | Type of outcome | | Time at which the discontinuation happened | Status (1= 1^st^ year discontinuer, 2= censored) | If discontinued, reason? |
| --- | --- | --- | --- | --- | --- | --- | --- | --- | --- | --- | --- | --- | --- | --- | --- | --- |
| 1 |  |  |  |  |  |  | |  |  |  |  |  | |  |  |  |
| 2 |  |  |  |  |  |  | |  |  |  |  |  | |  |  |  |
| 3 |  |  |  |  |  |  | |  |  |  |  |  | |  |  |  |
| 4 |  |  |  |  |  |  | |  |  |  |  |  | |  |  |  |
| 5 |  |  |  |  |  |  | |  |  |  |  |  | |  |  |  |
| 6 |  |  |  |  |  |  | |  |  |  |  |  | |  |  |  |
| 7 |  |  |  |  |  |  | |  |  |  |  |  | |  |  |  |
| 8 |  |  |  |  |  |  | |  |  |  |  |  | |  |  |  |
| 9 |  |  |  |  |  |  | |  |  |  |  |  | |  |  |  |
| 10 |  |  |  |  |  |  | |  |  |  |  |  | |  |  |  |
| **Period of insertion code:**   1. Post abortion 2. Immediate postpartum 3. Interval | | | | | | | **Type of outcome code:**  1. Unplanned discontinuation before 1^st^ year of use  2. Removal after 1 year of use  3. Removal due to desire to become pregnant  4. Switching after removal | | | | | | **If discontinued/removed/switched, reason code:**  1. Side-effects  2. Husband influence  3. Contraceptive failure  4. Misconception  5. Partner death | | | |

**Part II: Reproductive health, Implanon use follow-up, and outcome (A checklist used to extract from secondary data sources)**
