# Supplementary material for: Incidence and predictors of first-year unplanned discontinuation of Implanon at Ayder comprehensive specialized hospital, northern Ethiopia: A retrospective follow-up study
Source: PLoS One. 2022 Jan 26;17(1):e0259234. doi: 10.1371/journal.pone.0259234 (PMC8791466; doi:10.1371/journal.pone.0259234)
Supplement: S2 Table — (DOCX) [file pone.0259234.s002.docx]

**ማሕበራዊ ኩነታትን ስነ ህዝብን ዝምልከት ናይ ስልኪ ቃለ መሕተት (Tigrigna Version)**

ንስኺ ካብተን ኣብ መንጎ ሚያዝያ 2016 እና መጋቢት 2017 አቆጻጽራ ኣውሮፓውያን ኢምፕላኖን ዝኣተወን ሓንቲ ኢኺ። ስለዚ እዞም ዝስዕቡ ማሕበራዊ ኩነታት ዝምልከቱ ጥያቄታት ኢምፕላኖን ኣብ ዘእተውኽሉ ግዜ ዝነበረኪ ኩነታት ዘመልኽቱ ክኾኑ ኣለዎም።

| ተ.ቁ | ሕቶ | መልሲ | ናብ ዝቅፅል ይሕለፋ |
| --- | --- | --- | --- |
| 101 | ደረጃ ትምህርትኺ ክንደይ ነይሩ? | 1. ዘይተምሃረት 2. 1^ይ^ ብርኪ ዘጠናቀቀት 3. 2^ይ^ ብርኪ ዘጠናቀቀት 4. ኮልጅን ልዕሊኡን |  |
| 102 | ኩነታት ሓዳርኪ እንታይ ይመስል ነይሩ? | 1. ዘይተመርዐወት 2. ባዓልቲ ሓዳር 3. ዝተፋተሐት 4. በዓል ገዝኣ ዝሞታ | ዘይተመርዓወት እንተኾይና፣ ናብ ተ.ቁ 104 ይሕለፉ |
| 103 | በዓልቲ ሓዳር እንተነይርኺ ናይ ሰብኣይኺ (መፃምድኺ) ደረጃ ትምህርቲ ክንደይ ነይሩ? | 1. ዘይተምሃረ 2. 1^ይ^ ብርኪ ዘጠናቀቀ 3. 2^ይ^ ብርኪ ዘጠናቀቀ 4. ኮልጅን ልዕሊኡን |  |
| 104 | ስራሕኺ እንታይ ነይሩ? | 1. መዓልታዊ ሰራሕተኛ 2. ሰራሕተኛ መንግስቲ 3. ናይ ግሊ ስራሕ 4. ተምሃሪት 5. ሓረስታይ 6. ዘቤት ወይዘሮ 7. ካሊእ(ይግለፃ) |  |
| 105 | ናይ ሰብኣይኺ (መፃምድኺ) ስራሕ እንታይ ነይሩ? | 1. መዓልታዊ ሰራሕተኛ 2. ሰራሕተኛ መንግስቲ 3. ናይ ግሊ ስራሕ 4. ሓረስታይ 5. ካሊእ(ይግለፃ) | ንዘይተመርዐወት ጥያቄ ይሕለፋ |
